# General aptitude and the assumption of truth in deductively rational reasoning about probable but false antecedent to consequent relations

**DOI:** 10.2478/v10053-008-0079-z

**Published:** 2010-12-15

**Authors:** Walter Schroyens, Lieve Fleerackers, Sunile Maes

**Affiliations:** Laboratory of Experimental Psychology, University of Leuven, Belgium

**Keywords:** rationality, reasoning, conditionals, truth

## Abstract

Two experiments (*N*_1_ = 117 and *N*_2_ = 245) on reasoning with knowledge-rich conditionals showed a main effect of logical validity, which was due to the negative effect of counter-examples being smaller for valid than for invalid arguments. These findings support the thesis that some people tend to inhibit background inconsistent with the hypothetical truth of the premises, while others tend to abandon the implicit truth-assumption when they have factual evidence to the contrary. Findings show that adhering to the truth-assumption in the face of conflicting evidence to the contrary requires an investment of time and effort which people with a higher general aptitude are more likely to do.

## General Introduction

In recent discussion on the psychology of human thinking and reasoning it has been argued that theories that have focussed on reasoning under certainty (i.e., deductive reasoning) are incapable of being extended to reasoning under uncertainty (i.e., probabilistic reasoning). The “core argument” (Oaksford & Chater, 1998) is that common-sense reasoning is non-monotonic, whereas logic systems are monotonic: Once an inference is made that is logically valid, this inference remains logically valid. The validity of everyday inferences however would be revisable. For instance, almost everybody will at first accept the following so-called *Modus Ponendo Ponens argument* (MP): *If it is a bird, then it can fly; Tweety is a bird and, therefore, can fly*. At the same time, when subsequently being told that Tweety is an ostrich, almost everybody will reject the original inference and will state that Tweety cannot fly. Such a presumed revision of the validity of an argument is beyond the scope of standard monotonic logics.

Schroyens (2004, 2009, in press) argued however that some controversies seem to be non-issues, which could have been avoided in the first place by considering the distinction between the *defeasibility* and *non-monotonicity* of an inference (see also Politzer & Braine, 1991). The double meaning of *inference*, as referring to both the entailment relation and that which is entailed, that is, the conclusion, has most likely contributed to the conceptual confusions. *Monotonicity* concerns the validity of inferences; *defeasibility* concerns the truth of conclusions and this “distinction between validity and truth … is basic to deductive logic [and] many people find the distinction difficult to grasp” (Glass & Holyoak, 1986, p. 338). The truth-value of a validly inferred conclusion is always a hypothetical truth, whereas the truth-value (i.e., falsity) of a defeated inference hinges on a factual truth, that is, our belief, at a particular moment in time and space that something is true in the “real” world. Though they are closely linked, defeasible and non-monotonic inferences are not the same: The defeasibility of conclusions is a necessary, but not a sufficient condition for the non-monotonic nature of the arguments yielding that conclusion. Consider again our example of Tweety the flying ostrich.

The conclusion of the argument is false, but it cannot be rejected on logical grounds … What is wrong, of course, is that the claim that all birds can fly is true (Nickerson, 1986, p. 10).

The present study investigates the importance of the truth-assumption and the hypothetical nature of the truth of validly inferred conclusions.

Research on meta-propositional reasoning about the truth or falsity of propositional utterances (e.g., Rips, 1989, 1990) already provided evidence for the thesis that people start reasoning on the basis of the assumption that given information is true (see e.g., Schroyens, 1997; Schroyens, Schaeken, & d’Ydewalle, 1996, 1999). The following is an example of such meta-propositional puzzles (akin to the well-known liar paradoxes): *Walter says that if he speaks the truth then Jonathan is lying. Jonathan says: Walter is a liar*. What is the status of Jonathan and Walter? Are they liars or truth-tellers? The type of errors people make and the ease of solving the meta-propositional reasoning puzzles are in line with the truth-assumption, which Schroyens (1997) and Schroyens et al. (1996, 1999) have referred to as the *Gricean hypothesis*:

Rips (1989, 1990), Johnson-Laird, and Byrne (1990, 1991) suppose that subjects start solving such knight-knave problems by making a hypothesis about the truth-status of one of the assertors in the problem. Moreover, they all ho1d the view that this starting hypothesis generally is one whereby it is assumed that the person first mentioned in a problem is a truth-teller, which is in accordance with the maxims of Grice (1975). (Schroyens et al., 1996, p. 146)

Grice (1975) formulated his general ”cooperative principle” for conversation and, echoing Kant’s synthetic a priori categories specified his cooperation maxims of Quality, Quantity, Relation, and Manner. Truth regards the Quality of a contribution that would follow “the supermaxim ‘Try to make your contribution one that is true’ and two other more specific maxims: 1. Do not say what you believe to be false; 2. Do not say for which you lack adequate evidence” (p. 46). Though performance on meta-propositional reasoning problems evidences the psychological reality of a Gricean truth-assumption, it does not inform us about the relation between deductive or logical validity and hypothetical reasoning under the assumption of truth.

Other studies have provided some insight into the relation between the truth-assumption and logical validity. Markovits (1995; see also Markovits & Schroyens, 2007; Markovits et al., 1996) confronted his participants with contrary-to-fact conditionals (e.g., “If I throw the feather into the window, it will break”) that were sometimes presented in a fantasy context. The fantasy context conveys a hypothetical world, and stimulates as such a hypothetical mode of thinking that allows one to dissociate factual knowledge (about our world) from hypothetical knowledge (in some other imaginary world enunciated by language). When the clearly false conditionals were presented in a fantasy context, the children were indeed more inclined to accept the logically valid MP arguments (as well as the Modus Tollens [MT] arguments introduced below). This shows that stimulating a hypothetical line of reasoning under the assumption that something is true increases deductive rationality. Deduction presumes such hypothetical reasoning under the hypothetical-truth assumption. The truth of deductively valid arguments is thus always relative and never an absolute: “The deductions, in so far as they result from a correct process of reasoning, possess absolute validity only in reference to the same system of concepts to which the premises apply” (Shelton, 1912, pp. 80-81).

Given the centrality of the Gricean truth-assumption and the hypothetical nature of a conclusion’s truth in the notion of *logical validity*, we focus on the hypothesis that, at least to start with, people spontaneously make the assumption that the information they are given is true. The truth-assumption is a necessary component of deductively rational behaviour. Hence, if no evidence can be found that supports it, the idea that people can be (but do not need to be, cf. General Discussion section) deductively rational seems untenable. To investigate the Gricean assumption of truth we make use of well-known content effects (a.k.a. *belief bias*) in conditional reasoning. We first introduce these content effects.

### Content effects and the truth of an utterance

Table 1 presents the most commonly studied conditional inference problems. These problems are formed by an affirmation or denial of the antecedent (A) or consequent (C) of the conditional utterance of the form *if A then C*. The propositional content of the conditional utterance can be almost anything, for example:

*1. If you turn the key, then the car will start.*

*2. If you heat water to 100°C, then it will boil.*

*3. If you push the brake, then the car will stop.*

*4. If you jump into the swimming pool, then you’ll get wet.*

The content effects with such realistic conditional-inference problems show that the reasoning process is strongly affected by the factual truth of the premises and/or conclusion (Politzer & Bourmaud, 2002; see also Byrne, 1989; Cummins, Lubart, Alksnis, & Rist, 1991; De Neys, Schaeken, & d’Ydewalle, 2003).

**Table 1.** Standard Logically Valid or Logically Invalid Arguments About Conditionals of the Form “If Antecedent (A) then Consequent (C).”

Nomenclature		Argument	Counterexample
Logically valid			
Modus Ponens	MP	A therefore C	A and **not-C**
Modus Tollens	MT	Not-C therefore Not-A	Not-C and **A**
Logically invalid			
Affirmation of the consequent	AC	C therefore A	C and **Not-A**
Denial of the Antecedent	DA	Not-C therefore Not-A	Not-A and **C**

*Note*. The counterexamples to the inferences are formed by the categorical premise in combination with the denial of the conclusion (in bold).

The most robust content effects are *counter-example effects*. They reflect the effects of the number (and/or salience) of factual counter-examples to the standard inferences. For instance, the conclusions for AC and DA (cf. Table 1) are falsified by situations that reflect the possibility that the antecedent is false (*not A*) while the consequent is nonetheless observed (C). When the conditional captures a causal statement, such *not A and C* cases reflect so-called *alternative causes*. For instance, when people generate alternatives for conditionals (1) and (2), they generally come up with relatively few of them as compared to the number of alternatives for conditionals (3) and (4). The conclusions of MP and MT are countered by situations that represent the contingency where A is satisfied whereas C is not. When the conditional enunciates a causal statement, such A and not C cases reflect exceptions to the rule (a.k.a. *disabling conditions* or *disablers*, which affect whether the antecedent is a sufficient condition for the consequent to be the case). When people generate exceptions to the rules (1) and (3), they come up with a relatively high number of factors that might prevent the effect from occurring. For conditionals (2) and (4) there are only few exceptions. The most robust finding in reasoning with realistic, causal conditionals is that people are less likely to accept MP/MT when there are many exceptions and are less likely to accept AC/DA when there are many alternatives. The hypothesis that people make the Gricean truth-assumption has some straightforward implications as regards the counter-example effects tested in Experiments 1 and 2.

## Experiment 1

The present study investigated the relative size of counter-example effects on logically valid versus invalid arguments. The Gricean truth-assumption implies that counter-example effects should be smaller for the valid as compared to the invalid arguments. If a conditional is taken to be true, the True-antencedent-False-consequent (TF) cases are impossible. This is not a matter of debate “All theories of the conditional agree that the only state of affairs that contradicts *if the cat is happy then she purrs is a happy cat not purring* (TF), and so all other cases are possible” (Evans, 2007, p. 54). Meta-analyses (Schroyens, 2010) firmly establish that TF cases are judged impossible or are judged to show a conditional rule is false. These same meta-analyses also establish that False-antecedent-True-consequent, FT cases are often judged possible when people are reasoning about possibilities given that the conditional rule is true. Hence, for these cases there is no conflict between the Gricean truth-assumption and specific background knowledge about FT (not-A and C) cases (a.k.a. *alternative causes* or, in short, *alternatives*). It follows that the counter-example effect for the invalid arguments (no conflict for FT) would be larger than the counter-example effect for the valid arguments (a conflict for TF).

Though many studies have looked at the effect of reasoning about knowledge-rich conditionals with few versus many exceptions and/or alternatives, it is striking to see that as far as we know no study ever made a direct comparison between size of the counter-example effects on the valid and invalid arguments. This type of interaction between logical validity and belief is indeed a robust phenomenon in the literature on syllogistic reasoning (i.e., reasoning about subject-predicate expressions of the form *All A are B, No A are B, some A are /not/ B*; see e.g., Evans, Newstead, & Byrne, 1993) and it has been used to conclude that reasoning cannot be completely belief-based. If conditional reasoning similarly shows an interaction between logic and belief, then this poses problems for probabilistic theories of conditional reasoning that reject the psychological reality of the distinction between logically valid versus invalid arguments and propose that reasoning is largely if not solely belief based. The conditional-probability theory indeed rejects the idea that people make the Gricean truth-assumption.

If people adhere to the truth-assumption, they need to inhibit background knowledge in the context of the valid arguments. Such an inhibitory process or conflict resolution is likely to put demands on limited processing resources (see e.g., Engle, Conway, Tuholski, & Shisler, 1995; Gorfein & Macleod, 2007). We thus expected that people with higher ability would be more able to do this. That is, first we expected to observe larger counter-example effects on the invalid versus valid arguments. Second, the smaller effect of many versus few counter-examples on the valid arguments yields a main effect of logical validity. Third, the logical validity effect would be modulated by participants’ general ability. Participants with higher general ability would be more able to inhibit background knowledge and would thus be less likely to reject the logically valid arguments. Since there is no need to inhibit background knowledge in the case of the invalid arguments (as there is no conflict between the consequences of making the truth-assumption and this background knowledge), one does not expect general ability to modulate the logically invalid arguments. This holds provided that general ability is related to inhibition and is by itself not related to a larger knowledge base of potential counter-examples, that is, alternatives to the antecedent-to-consequent relation described in the conditional. But, this nuance does not affect the predicted interaction. If it is related to knowledge about alternatives, then general ability would also be positively related to a larger knowledge base of exceptions, which would need to be inhibited when following up the truth-assumption’s consequences in the context of valid arguments (but not the logically invalid arguments).

Being able to inhibit background knowledge obviously does not imply one actually makes the effort of doing so (e.g., many people are capable of killing another person, but luckily enough this does not mean they do it). However, if we observe that people with higher ability actually inhibit background knowledge, then this presumes at least these people were actually attempting to do so. The increased size of the logical-validity effect would thus provide converging evidence for the thesis that in a communicative context like the one between an experimenter and participant, people spontaneously make the assumption that speakers are providing true information by uttering the claims they make (Grice, 1975).

### Method

#### Participants

Participants were 11th- and 12th-grade students (*N* = 117) at a secondary Flemish high school within the general education system preparing for higher education.

#### Material, Design, and Procedure

Participants received a set of conditional inference problems with few or many counter-examples. The problems were either logically valid (MP, MT) or logically invalid. Participants were classified as being of low, medium, or high aptitude on the basis of their raw scores on the Standard Raven Progressive Matrices.

The conditional-inference problems were part of a larger battery of reasoning problems investigated to address other research questions. Participants first solved a set of 16 abstract propositional-reasoning problems about *if, only if, or else*, and *unless*. They then solved a set of 12 abstract spatial-relation problems (e.g., “The pear is to the right of the kiwi, the kiwi is to the left of the tomato, the apple is in front of the kiwi, the lemon is in front of the tomato: What is the spatial relation between the apple and the lemon?”). For the purposes of the present study, these problems are considered filler items. The 11th- and 12th-grade students are a subset of the complete number of participants. They served as the reference group for the study of developmental effects. That is, the entire study was run at all age high-school grades. The development of human reasoning falls beyond the scope of the present study and is not discussed here.

Participants evaluated 32 arguments (MP, MT, AC, or DA), presented in the following format (translated from Dutch):

Rule: *If John lies in the sun for a long time, then his skin will get burned*.

Fact: *John lies in the sun for a long time*.

Conclusion: *John’s skin is burned*.

The arguments were formed on the basis of eight knowledge-rich conditionals for which pilot studies have shown that they yield many or few disablers and/or alternatives (see Verschueren, Schaeken, & d’Ydewalle, 2005). The specific conditionals were taken from De Neys et al. (2002, 2003; cf. Appendix A), who classified the conditionals as having few versus many alternatives and/or exceptions on the basis of a separate study. The problems were introduced as follows:

We are interested in seeing how people reason with ordinary sentences. In each of the following problem you are given a general rule and a fact. A conclusion is derived from this rule and given fact. It is your task to evaluate the conclusion. For each problem you have to indicate how certain you think it is that the conclusion follows from the rule and the given fact.

Participants evaluated the conclusion on a symmetrical 7-point scale, ranging from *very/somewhat uncertain to somewhat certain, certain*, and *very certain*. The scale was repeated with each of the numbered problems and participants crossed the appropriate column (A, B, etc. up to G) for the respective problems on a special-purpose answer sheet. The study was run in two sessions in the individual classrooms. During the first session, participants solved the Standard Raven Progressive Matrices (SRPM). The second session took place about a week later.

### Results and discussion

**Figure 1.**
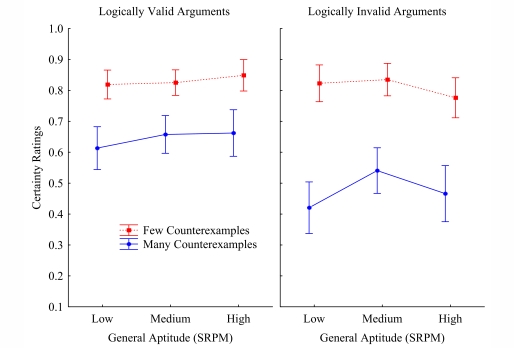
Argument-certainty ratings on the logically valid versus invalid arguments as a function of general aptitude, and counterexample frequency (few vs. many; Experiment 1).

The certainty ratings (1-7) were transformed to the [0, 1] probability interval and submitted to analyses of variance on the mean certainty ratings on the logically valid versus invalid inferences with few versus many counter-examples (see Figure 1). The counter-example effects reflect the effect of conditionals with many versus few alternatives on the logically invalid inferences (averaged over AC and DA), and the effect of many versus few exceptions on the logically valid inferences (averaged over MP and MT). This implies that for the valid arguments one averages across the frequency of alternatives, while for the invalid arguments one averages across the frequency of exceptions to the rules. For future reference in meta-analyses on the non-counter-example effects, Appendix B (Table B1) presents the full set of results. A between-groups factor was formed by general ability, as measured by the Standard Raven Progressive Matrices. For 12 participants no SRPM score was obtained during Session 1. These participants, as well as five participants who had not solved all problems, were excluded from the analyses. The remaining 100 participants were split into three general aptitude groups (low: *n* = 32; medium: *n* = 41; high: *n* = 27) on the basis of the 33rd (SRPM = 54) and 66th percentile (SRPM = 58). The boundary cases with SPRM 54 and 58 were placed in the medium group. Other studies (see e.g., De Neys et al, 2005) have selected participants on a similar basis to increase the contrast between low and high ability, without even retaining the medium ability group. The maximum raw score of the SRPM is 60. The present subjects group showed a relatively high mean score of 54.54 (*SD* = 4.51).

Figure 1 presents the mean certainty ratings as a function of logical validity, counter-example frequency, and general aptitude. It shows, first, the well-known counter-example effects, *F*(1, 97) = 258.1, *MSE* = .026, *p* < .000001. Both the valid and the invalid arguments were evaluated as less certain when there were many (vs. few) counter-examples to the conclusions, .831 vs. .644, *F*(1, 97) = 133.3, *MSE* = .013, *p* < .00001; and .811 vs. .486, *F*(1, 97) = 228.8, *MSE* = .024, *p* < .00001, respectively. More interesting for the present discussion is the interac tion between logical validity and counter-example frequency, *F*(1, 97) = 49.2, *MSE* = .011, *p* < .00001. Figure 1 shows that the counterexample effect on the valid arguments (.831 vs. .644: *d* = .187) is smaller than the counterexample effect on the invalid argument (.811 vs. .486: *d* = .325). This finding corroborates the hypothesis that people make the Gricean truth-assumption and consequently inhibit factual knowledge that conflicts with implications of this assumption about cases that are (im)possible.

We conjectured that people higher in general ability might be more apt to inhibit background knowledge when such is needed. The interaction between general aptitude and logical validity did not reach statistical significance at the conventional level, though there was a strong tendency *F*(2, 97) = 2.8, *MSE* = .019, *p* = .063. A statistically more powerful test does not hinge on testing an interaction between the decreased certainty ratings of the invalid arguments and the increased certainty ratings of the valid arguments. The opposing effects are in the same direction if one were to use logically correct responding as the dependent measure, which is a delicate matter since the label *correct* reflects an evaluative and/or normative stance towards human reasoning performance. In the following we will continue to use the term *logically correct* to annul the possible connotation that such *correct* behaviour would be normative. It is only correct relative to the standard of classic logic, and this standard (like any norm) is a non-absolute that is open for discussion (Schroyens, in press). In the case of the invalid arguments one could use the complement of the certainty rating as a measure of logical correctness. An equivalent procedure to evaluate the overall relation between logical correctness and general ability consists in computing a logic index as the difference between the certainty ratings of the valid versus invalid arguments. There is a positive correlation with general ability (*r* = .135). It did not reach statistical significance though (*p* = .182).

## Experiment 2

Experiment 1 confirmed that the counter-example frequency effect is smaller in the context of valid versus invalid arguments. This finding is consistent with (i.e., derives from) the Gricean truth-assumption. The assumption’s implication that exceptions (i.e., TF cases) are impossible if a conditional were true, conflicts with background knowledge about the factually possible exceptions to the rule. Some people seem to adhere to the truth-assumption and inhibit the conflicting background knowledge. This consequently results in smaller counter-example frequency effects on the valid arguments as compared to the invalid arguments for which there is no such conflict (at least providedthe conditional is not interpreted as the bi-conditional *if and only if*).

Of course, the fact that there remains a reliable counter-example effect on the valid arguments shows that certainly not all people limit the problem space to the narrow confines of the possibilities delineated by assuming the conditional is true. The counter-example effect on the valid arguments (even though smaller than on invalid arguments) demonstrates that many people abandon the truth assumption and take their broader background knowledge into account to judge the certainty of the arguments.

Experiment 1 only yielded suggestive but not conclusive (i.e., statistically significant) evidence for a positive relation between general aptitude and logically correct performance. Though the results were in the expected direction, subjects with higher ability did not show a statistically reliably larger effect of logic (i.e., the truth assumption) by showing increased certainty ratings of the valid arguments and/or showing decreased certainty ratings of the invalid arguments. Experiment 1 used a relatively selective sample, though. All participants were students in private colleges, that is, non-public secondary high schools that do not provide technical education but provide general education in preparation for higher education. The present study tried to remedy the restriction in the range of general aptitude by sampling from different educational systems (i.e, both technical and general). Moreover, the Standard Raven Progressive Matrices does not distinguish well in the higher regions of general aptitude. We therefore decided to use a more extended battery of tests, using both measures of fluid and crystallised intelligence to obtain a measure of general aptitude.

### Method

#### Participants

Participants were 11th- and 12th-grade students (*N* = 245) at a secondary Flemish high school. Participating high schools were of two types. They either provide technical education or else provide general education in preparation of higher education. For both the 11th and 12th grade, one class was drawn from a school for technical education and one class was drawn from a school for general education.

#### Design

Participants received logically valid (MP, MT) or invalid (AC, DA) conditional inference problems with few or many counter-examples. A first between-groups factor was formed by a measure of general aptitude (low, medium, high). A second between-groups factor was formed by inviting participants to provide their evaluation of the conclusions asap (*n* = 116) or not stressing them (*n* = 129).

#### Materials

##### Conditional-inferences problems

Participants evaluated the same 32 conditional arguments used in Experiment 1. The conclusions were evaluated on the following 5-point scale, ranging from *very/somewhat uncertain* to *somewhat/very certain that the conclusion follows*. The scale was repeated on the right-hand side of each of the numbered problems and participants ticked their response (A, B, etc) to this problem on a separate response sheet.

As in Experiment 1, the conditional-inference problems were part of a larger battery of reasoning problems. Before solving the problems of interest for the present study, participants first solved a set of 32 syllogisms (i.e., problems based on premises with *all, none, some*) with believable or unbelievable conclusions. As in Experiment 1, the 11th- and 12th-grade students formed the young-adult reference group for a study in the development of human reasoning, which is a topic of interest that falls beyond the scope and focus of the present study and will be not discussed here.

About half the participants were invited to solve the problems as soon as possible (119 of the 249 11th- and 12th-grade students). Everything was identical to the non-speeded group, except that the speeded group read the following additional paragraph in the instruction to the different reasoning problems:“You have to try to solve the problems AS FAST AS POSSIBLE. This does not mean that you can fill in just anything. You have to select the answer you think is correct, but as fast as possible. This test more particularly probes for your fast, initial ‘gut-response’ judgements on the problems.” The speeded-inference instructions were added for exploratory purposes (but see Schroyens, Schaeken, & Handley, 2003, for a rationale of using speeded-inference).

##### Psychometric tests

Participants answered three sub-tests (Analogies, Figures, and Words) of the Dutch Differentiële Aanleg Test (Differential Aptitude Test series, D.A.T.; Evers & Lucassen, 1991). The Analogies sub-test consists of 50 sentences of the following type “… stands for sweet such as lemon stands for … (a) school – car, (b) work – hotel, (c) sugar – sour, (d) wood – fork, (e) eating – breakfast.”

The Figure-Series test is analogous to the Raven Progressive Matrices and consists of 50 items. The Words test probes for the meaning of 75 words. Participants are given a target word and have to select among a list of five answer alternatives the word that most closely matches the target word’s meaning. Participants also completed the Rationality-Experientiality inventory (REI; Pacini & Epstein, 1999). The REI consists of two sub-scales (Rationality, i.e., the original Need for Cognition scale, and Experientiality) which are measured by 20 items (assertions) each. Participants have to indicate to what extent (1-5) they consider the assertions applicable to themselves, for instance “I generally prefer to accept things as they are rather than to question them.” The psychometric tests were completed in a 2-hr session and were passed in a fixed order and within a fixed time limit (25 min for the Figure Series, 20 min for the Analogies and 20 min for the Words test). The remaining time (15 min) was left to complete the REI.

### Results and discussion

**Figure 2.**
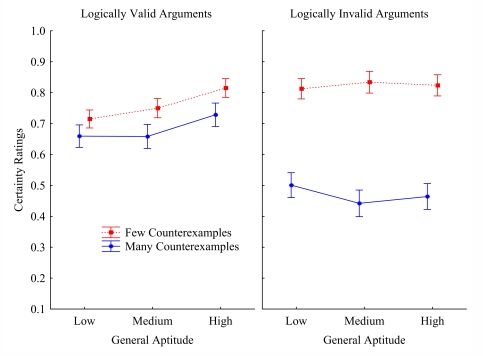
C ertainty ratings as a function of logical validity, counterexample frequency (few vs. many) and general aptitude in Experiment 2.

The certainty ratings (1-5) were transformed to the [0, 1] probability interval and submitted to analyses of variance on the mean certainty ratings on the logically valid versus invalid inferences with few versus many counter-examples (see Figure 2). General aptitude (low, medium, high; as determined by the 33rd and 66th percentile, cf. Experi-ment 1) was introduced as a between-subjects variable in the ANOVA. The general aptitude score was computed as the proportion of correct responses to the Analogies, Figure Series, and Words tests. An equal weight was given to each of the three sub-tests. Table 2 presents the correlations between the different sub-tests, as well as the correlations with the Logic Index. This index is computed as the difference between the certainty ratings of the valid minus the invalid arguments. It thus corresponds to the logical-validity effect in the ANOVA. Preliminary analyses showed that the speeded-inference instruction did not show a main effect and did not interact significantly with any of the other variables (both in first, second, or third-order interactions).

**Table 2.** Correlations Between Reasoning Performance Metrics and Metrics of General Ability Metrics and Cognitive Style (Experiment 2, N = 245).

	Valid		Invalid		R		E		G		Words		Analogies		Figures	
	*r*	*p*	*r*	*p*	*r*	*p*	*r*	*p*	*r*	*p*	*r*	*p*	*r*	*p*	*r*	*p*
Logic index	.437	.0007	-.509	<.0001	.172	.0072	-.157	.0146	.340	.0009	.283	.0002	.327	.0007	.227	.0007
Percentage valid			.552	<.0001	.176	.0066	-.021	.7500	.227	.0006	.167	.0096	.220	.0019	.164	.0104
Percentage invalid					.009	.8879	.126	.0505	-.098	.129	-.102	.1122	-.093	.147	-.053	.4063
Rationality (R)							-.000	.9980	.267	.0006	.243	.0003	.292	<.0001	.116	.0706
Experientiality (E)									-0,73	.254	-.102	.113	-.089	.167	.003	.9632
G											.724	<.0001	.929	<.0001	.788	<.0001
Word lists													.623	<.0001	.340	.0009
Analogies															.569	<.0001

*Note*. G is formed as the weighted sum total of scores on the Word-meaning, Analogies, and Figure-series tests.

Figure 2 presents the mean certainty ratings as a function of logical validity, counter-example frequency, and general aptitude. As in Experiment 1 (see Figure 1), Figure 2 clearly shows the well-known counter-example effects, *F*(1, 239) = 665.7, *MSE* = .0168, *p* < .000001. Both the valid and the invalid arguments are evaluated as less certain when there were many (vs. few) counter-examples to the conclusions, 760 vs. .681, *F*(1, 239) = 70.3, *MSE* = .010, *p* < .000001; and .823 vs. .469, *F*(1, 239) = 944.1, *MSE* = .0159, *p* < .000001, respectively. More interesting for the present discussion is the interaction between logical validity and counter-example frequency, *F*(1, 239) = 484.1, *MSE* = .0094, *p* < .00001. Figure 2 shows that the counter-example effect on the valid arguments (.760 vs. .681: *d* = .079) is again smaller than the counter-example effect on the invalid argument (.823 vs. .469: *d* = .354). This concurs with the hypothesis that at least to start, people make a truth-assumption. As before, though people might start with the truth-assumption, the significant counter-example effect on the valid argument (*d* = .079 irrespective of it being smaller than in the invalid arguments) indicates that people often do not maintain the truth-assumption. They often seem to abandon the truth-assumption in favour of taking background knowledge into account. Not always though, as indeed the counter-example effect is significantly smaller on valid versus invalid arguments.

Experiment 1 attempted to provide converging evidence for the primacy of the truth-assumption by relating ensuing conflict resolution to general aptitude. It was hypothesized that participants with a higher general aptitude are more apt to resolve the conflict by inhibiting background knowledge that is inconsistent with the truth-assumption. When assuming the conditional is true, exceptions to the rule are judged impossible. That is, the counterexamples to the valid arguments (i.e., the exceptions to the rule, a.k.a. *disablers*) are inconsistent with the truth-assumption. Figure 2 confirms the positive relation between general aptitude and logically correct reasoning, which is by definition reasoning on the basis of the truth-assumption. Table 2 indicates that there is a significant positive correlation between the logic Index and General aptitude, *r* = .34, *p* < .0001. The analyses of va-riance accordingly yield a significant second-level interaction between general aptitude and the certainty ratings of the logically valid versus invalid arguments, *F*(2, 239) = 11.5, *MSE* = .0172, *p* < .00001. Table 2 similarly shows a positive relation between Rationality (a.k.a. *need for cognition*) and deductive rationality, that is, the logic index. People who score high on the Rationality index are more likely to endorse the logical valid arguments. Endorsing these arguments involves resolving a conflict between the exceptions being (hypothetically) impossible while background knowledge informs us they are (factually) possible.

Figure 2 shows that people with a higher general aptitude are less likely to reject the logically valid arguments, *F*(2, 239) = 8.6, *MSE* = .038, *p* < .001, whereas general aptitude does not affect certainty ratings of the invalid arguments, *F* < 0.5. This is exactly what one expects when inhibition is related to general ability and background knowledge needs to be inhibited in the context of the valid arguments but not the invalid arguments. The absence of a general-ability effect on the invalid arguments suggests that it is not knowledge or access to background knowledge that is positively related to general aptitude. If this were the case than high ability people would show lower acceptability rating of the invalid arguments. And if it were (which seems plausible at first sight, cf. Verschueren et al., 2005), then this makes the theoretical import of a positive effect on the valid arguments even stronger. It would mean that despite increased knowledge of counter-examples and/or increased ability to retrieve counter-examples the high-aptitude reasoners discount their larger knowledge base when it conflicts with the truth- assumption. The present findings thus suggest that general aptitude is not directly related to increased knowledge but is related to what one does with this factual knowledge. The relation between the Rationality subscale of the REI and logically valid reasoning (see Table 2) converges upon this conclusion. Having a particular competence is almost useless if one does not use it. This is trivial when phrased as such (use-less, vis-à-vis, non-use). One must also be motivated to adopt and develop one’s talents and capacities in order to fulfil one’s potential. The Rationality Index taps into such a motivational need for cognition. Overall, findings are consistent with the central thesis that some people inhibit factual background in cases where it conflicts with the spontaneous assumption that given information is truthful.

## General Discussion

The present study investigated the importance of a Gricean truth-assumption as regards the language game of reasoning under certainty, that is, deductive rationality in human reasoning. Both studies presented evidence in favour of the Gricean truth-assumption. First, both studies showed smaller counter-example effects on the valid versus invalid, which lies at the basis of the main logical validity effects. The valid arguments are more likely to be endorsed than the logically invalid arguments, supposedly because following up the consequences of the hypothesized truth-assumption requires inhibition of the counter-examples to the valid but not the invalid arguments. Second, both studies provided suggestive evidence in favour of hypothesized inhibition of counter-examples. Such inhibition would be required in the context of the valid but not the invalid arguments and the results indeed showed that general ability (which makes execution of inhibitory processes easier and/or more likely) is positively related to the size of the logical validity effect.

Our predictions for the Gricean truth-assumption were derived and specified without relying much on the specific details of one or other processing theory. Given the available evidence, however, the implication for extant theories of reasoning are rather straightforward. Those theories that subscribe to the truth assumption seem strengthened, whereas theories that do not, seem confronted with a set of more difficult-to-explain findings. In the following two sections we give an example of these two types of truth versus truthfulness-based theories. We then touch upon some wider theoretical and conceptual issues. We first present a brief consideration of the notion of *truth* (*verity* or *strict truth*) as compared to *truthfulness* (*verisimilitude*). This distinction is fundamental to the contrast between extant alternative theories of reasoning about conditionals. We subsequently consider the rational basis for the truth-assumption and end the general discussion by briefly considering the notion of deductive rationality.

### Truth-based interpretations of conditionals

 Most current theories of human reasoning presume the truth-assumption. This is not very surprising when one considers that truth is primordial to falsity: Non-truth presumes truth. “Though Truth and Falsehood bee Neare twins, yet Truth a little elder is.” (Donne, 1635/1930, p. 129, cited in Gilbert, 1991). The mental-model theory (Johnson-Laird & Byrne, 2002) is the single one theory that is most explicit in invoking the truth-assumption (Johnson-Laird, 1983). It forms the basis of the truth-principle as regards the representation of conditionals. The truth principle states that “each mental model of a set of assertions represents a possibility *given the truth of the assertions”* (Johnson-Laird & Byrne, 2002, p. 653). This truth-principle is misrepresented when stating, as one sometimes sees claimed in the literature, that people only represent true possibilities to suggest that people initially only represent possibilities that make the conditional true. The truth-principle is not a categorical claim about cases that make the conditional true (i.e., when reasoning about the conditional). Johnson-Laird and Byrne (2002) are very explicit in claiming that truth judgements are distinct from judgements about possibilities: 

Each entry in a truth table represents the truth or falsity of an assertion given a particular possibility. In contrast, each mental model in a set represents a possibility. A corollary is that possibilities are psychologically basic, not truth values. Discourse about the truth or falsity of propositions is at a higher level than mere descriptions of possibilities. (p. 653)

The truth-principle is a conditional claim about what is represented as possible, when reasoning from conditionals, that is, when reasoning from the assumption that the conditional is true. The representation of possibilities (i.e., mental models of such possibilities) is conditional upon the assumption that the proposition is true. True possibilities are, by definition, the states of affairs that are possible, given that the proposition is true.

Mental-model theory proffers that by default people start reasoning from the assumed truth (vs. truthfulness) of a proposition. That is, using Gilbert’s (1991) classification, mental-model theory defends a “Spinozan system”. In Spinozan systems a strict belief in the truth of the conditional is the default. This strict belief can subsequently be “probabilified” (to use Morris & Sloutsky’s, 1998, term) by taking exceptions to the rule into account. In so-called ‘Cartesian systems’ it works the other way round. That is, a fuzzy probabilistic belief in the conditional is the default, though this subjective belief can be “upgraded” to strict belief P(*q*|*p*) = 1 by discounting exceptions to the rule. In the following section we illustrate how the present evidence in favour of the Gricean truth-assumption seems problematical for conditional-probability theories that proffer a Cartesian belief system in which it is assumed people start reasoning by default from their non-strict belief in the truthfulness (i.e., subjective probability) of the conditional.

### Truthfulness-based interpretations of conditionals

Conditional-probability theories (e.g., Evans & Over, 2004; Oaksford, Chater, & Larkin, 2000) are a class of theories that seem to have difficulty incorporating the Gricean truth-assumption. They do not seem to distinguish true from false utterances. There are only degrees of falsity or truth (i.e., probabilities). This restriction to factual truth (truthfulness or verisimilitude vs. truth or verity) is problematical because there is enough evidence showing that people can reason hypothetically and deductively. Schroyens and Schaeken (2003) have indeed shown that the conditional-probability model of conditional reasoning is deficient because it is purely probabilistic, that is, belief-based in nature (see also Oaksford & Chater, 2003).

An observation that is problematical for conditional-probability theories is that some people seem to make the truth-assumption without being instructed to do so. Moreover, our findings show that it is particularly people with higher general ability that seem more consequential in making the truth-assumption. The logical-validity effect that follows from the truth-assumption is observed even though reasoners are not instructed to reason logically and/or are not instructed to assume the conditional premise is true. This is an important difference with reasoning tasks that are explicitly deductive in nature. In such deduction studies participants are (and need to be; cf. Evans, 2002) instructed to assume the premises are true. Indeed, at first sight, conditional-probability theories have little difficulty in explaining an effect of stressing the truth-assumption in such deduction studies.

For instance, Schroyens (2004) instructed participants that they had to assume the conditional was true even if it might in fact not be strictly true. Under these conditions the logical-validity effect increased as compared to when there was no mention of assuming the conditional to be true. Phrased within the scope of conditional-probability theory, stressing the truth has the simple consequence that the subjective belief in the conditional *if p then q* (i.e., conditional-probability of *q*, given *p*) is set to 1: There are no exceptions to the rule. The normal contextual relativity of the conditional claims is blocked by imposing the truth-assumption. The effect of stressing the truth-assumption (Schroyens, 2004) is theoretically informative only to the extent that it shows that the truth-assumption has the predicted import on the logical validity effect and strengthens an effect that is also present when people are reasoning in a normal context that does not invite them explicitly to constrain their beliefs to an artificially created context. The smaller size of the counter-example effects on the valid versus invalid arguments suggests that people do not reason simply on the basis of factual knowledge and/or their subjective belief in the conditional. That is, the contextual relativity of conditionals does not seem to be primordial.

Consider the conditional-probability model (Oaksford et al., 2000) in which the MP and AC endorsement rates are a direct function of the conditional probability of the conclusion, given the categorical premise.

P(MP) = P(*q*|*p*)

P(AC) = P(*q*|*p*)

These functions are easily reformulated as a function of counter-examples:

P(MP) = 1 – P(~*q*|*p*)

P(AC) = 1 – P(~*q*|*p*)

Assuming that P(*q*|*p*) = P(*q*|*p*), it follows that P(MP) must be equal to P(AC). Both experiments show that this is not the case and that the MP rates are higher than the AC certainty ratings.

Of course, to undercut the falsified prediction, conditional-probability theory might rebut that the assumption is not satisfied and that P(*q*|*p*) is larger than P(*q*|*p*). This is possible, but certainly very unlikely given the experimental control of our studies. First, the stimulus materials used in Experiments 1 and 2 are closely matched on the saliency and frequency of *p* and ~*q* (a.k.a. *exceptions* or *disablers*) and ~*p* and *q* (a.k.a. *alternatives*). De Neys et al. (2002) already reported summary statistics indicating that the alternatives and exceptions we used in Experiments 1 and 2 are comparable in their average frequency, plausibility, and salience. (Salience is computed as the proportion of subjects who generate the most frequently generated alternative or exception.) The conditional probabilities are directly related to the likelihood of the exceptions and alternatives and, indeed, the conditional probabilities are not basic. As noted by Ohm and Thompson (2006): 

These probabilities, however, are not explanatory constructs. Rather, they are mathematical summaries that represent the culmination of one or more underlying representational processes. … For example … Thus, it seems likely that probability estimates may be mediated by the availability of instances of the form *p and ~q*, or *~p and q*, that come to mind. (p. 272)

Given the close matching of the availability of the *p and not-q* and *not-p and q* cases, though not impossible, there is thus certainly little room to argue P(*q*|*p*) has been systematically higher than P(*q*|*p*).

The logical validity effect, which is grounded on the smaller effect of counter-examples to the logically valid arguments, shows that the argument certainty ratings and/or endorsement rates are not merely a function of their conditional probabilities. Conditional-probability theories need to invoke additional processes to explain the findings. We argued that such processes are related to a Gricean truth-assumption that people would spontaneously make when given information they are invited to reason from. Assuming at least to start that given information is true, there is a conflict between the exceptions to the rule being impossible if the rule were true, on the one hand, and the exceptions to the rule being factual possibilities, on the other hand. At least some people seem to make the Gricean truth-assumption spontaneously. Making and adhering to the truth-assumption results in reducing the potential impact of exceptions to the rule. Indeed, when the rule is assumed to be true there are no exceptions to the rule.

The effect of the truth-assumption (i.e., inhibition of exceptions to the rule) is within the grasp of conditional-probability theories, at least apparently so. These theories have difficulty though in explaining why people seem to make the Gricean truth-assumption in the first place (i.e., why it is “Gricean” in nature). Indeed, probabilistic subjective-believability and not absolute truth is considered to be the default and primary in human reasoning. Moreover, the present results further constrain any amendment to conditional-probability theories in giving body to an algorithmic level specification of the simple (too simple) computational model proffered by Oaksford et al. (2000): Especially higher general-ability people seem susceptible to inhibiting background knowledge that is inconsistent with the hypothetical truth of the conditional one is reasoning from (vs. about).

### An implicit versus explicit truth-assumption

We found support for the thesis that at least some people make the truth-assumption and actually stick to it. The logical validity effect indicates that counter-examples to valid arguments are given less weight. It remains the case, however, that the majority of people will abandon the truth-assumption. The sizable counter-example effects one observes on the logically valid inferences evidence this. One can only claim that the truth-assumption is abandoned when it is made in the first place. The question that then arises is whether those people who do not follow the truth-assumption (by taking factual knowledge to the contrary into account) actually made it in the first place.

The idea that people initially and implicitly make the assumption that the proposition they are confronted with is true, is in accordance with the Gricean maxims of conversation: We generally assume/ensure that our or the speaker’s contribution is truthful, relevant and as informative as possible, though not more detailed than required by the context (Grice, 1975; see also Levinson, 2000). Or, as noted by Gupta and Belnap (1993):

In more recent times, Gottlob Frege, Frank Ramsey, and others have made the related observation that the sentence *that p is true* had the same meaning as *p* – that the addition of the truth predicate does not contribute any new content to the sentence *p*. (p. 1)

The truth-assumption is an implicit assumption (see e.g., Schroyens, 1997; Schroyens et al., 1996, 1999). It is partly because it is an implicit assumption that it is easily abandoned. The rational basis of the truth-assumption can be found in the idea of bounded rationality or cognitive economy. There is a representational cost attached to considering all possibilities, both true and false.

### Verity and verisimilitude

In the General Introduction section we suggested that defeating or suppressing a valid argument can simply mark the abandonment of the hypothetical-truth assumption (see also Politzer & Braine, 1991). Theorists arguing against (mental) logic theories contest that questioning the literal truth of, for example, *If it is a bird, then it can fly* is involved in defeasible reasoning: “Surely [this] mischaracterizes people’s cognitive attitude towards this and a million other commonsense generalizations” (Oaksford & Chater, 1998, p. 5). This rhetorical claim as regards the truthfulness of strictly speaking false conditionals misses its target because it is not congruent with reality. We ran an additional study to address this issue. We do not need to allocate much space to present this study in its usual format (i.e., laboriously and by giving a Method section with Procedure, Design…). Indeed, we simply asked 44 first-year psychology students to evaluate whether the conditional “If it is a bird, then it can fly” is “true or false,” while at the same time we told them – translated from Dutch – ”to think about the fact that for instance ostriches and penguins are also birds (and can not fly).” Thirty-eight of them (86%) judged the conditional to be false. In short, the factual falsity of the conclusion *Tweety the ostrich can fly* licenses the conclusion that *If it is a bird, then it can fly* is a false utterance.

To ground the core argument against “mental logic,” Oaksford and Chater (1998) appeal to the, for many people comforting, idea that there is true common-sense knowledge. 

If our commonsense descriptions of the world and of ourselves are not candidates for truth then precious little else of what we call our commonsense knowledge of the world will be candidates for truth. We would then be in the paradoxical position of having to provide a system of human inference that is always based on false premises but which is nonetheless apparently capable of guiding successful action in the world! (Oaksford & Chater, 1998, p. 5)

There is really only an apparent contradiction. It is not that problematical that there is precious little (if any) knowledge that is strictly true. The fact that some birds do not fly does not make it senseless to use the generalization that birds fly. An absolute truth is universally applicable, but if something is not universally applicable then this does not imply that the idea is inapplicable and useless. It might be inapplicable (applicable to none) or applicable to some (but not all). The demonstrable fact that most of our common-sense generalizations are false (i.e., not strictly true), marks that they only have a certain degree of truth: They are false, but applicable (or “assertible”; see Adams, 1975). *Verity* is not *verisimilitude*. Rips (2001) already highlighted that there is a world of possibilities between something being absolutely false (i.e., having a probability of 0) and being absolutely true (and having a probability of 1).

### Deductive rationality: Adaptively rational

The idea that people at least sometimes exhibit deductively rational behaviour has become a controversial thesis. In recent years, the first author has argued however that the “probabilistic turn” (Oaksford & Chater, 2007) is in danger of making an overturn, irrespective of it having provided a valuable contribution to the literature in correcting “logicist” preconceptions about what human rationality is about. It seems that the same theorists who critiqued so-called *mental logicians* for their “reasoning imperialism” (Rips, 2001) as regards deductive logic have become reasoning imperialists in advancing “mental Bayesianism” as the absolute standard of human rationality. We certainly do not defend a strong version of mental logic, but defend the thesis that deductively rational behaviour can be adaptively rational.

In our view (Schroyens, 2009, in press), *deductive rationality* is a form of adaptively rational behaviour (Anderson, 1990) where the human processing system adapts itself to the context and goals of deductive reasoning under certainty. The first step in a so-called *rational analysis* is indeed to “specify precisely the goals of the cognitive system” (Chater, Oaksford, Nakisa, & Redington, 2003, p. 69). Given the notion of *adaptively rational behaviour* – where, by definition, rationality is determined as a function of the context and current processing goals of the system – one can never use *rationality* in an absolute and unqualified sense. Rationality is relative to the adaptive context and processing goals of the inferential system. This also means that the observation that people *can* reason deductively does not imply the evaluative stance that people *should* (in an absolute, context-independent and non-relativistic sense) reason deductively and neither does it imply that people would always exhibit deductively rational behaviour in common-sense reasoning (see Mandel, 2000, for a discussion of “conceptual blur in the rationality debate”).

In common-sense reasoning about ordinary language expressions and beliefs such as *If Tweety is a healthy and mature bird, then Tweety can fly*, there are many practical issues that often prevent people from setting the goal of making deductively valid arguments. In other words, deductively rational behaviour is often very impractical. Critical thinking and reflectiveness does not always serve our daily purposes (see e.g., Baron, 1990; Duemler & Mayer, 1988; Holt, 1999; Shugan, 1980). It would be infelicitous to act only upon inferences that follow necessarily. Plausible or likely inferences, though not necessarily true, can be helpful in informing and guiding actions:

When faced with the ubiquitous sabre-toothed tiger of which armchair evolutionists are so fond, the reasoner does not want to hang around working through some hellish normative theory: He or she wants to act, and fast. On the other hand, back in the safety of the cave it makes sense to evaluate in a reliable and communicable way the consequences of a decision to act, so that the individual and his or her social group can learn from the event. (Ormerod, 1997, p. 183)

The present study set out to investigate the general thesis that human thinking and reasoning contains the seeds required to exhibit deductive rational behaviour. One central aspect of deductively rational behaviour is hypothetical reasoning under the assumption of truth. Experiments 1 and 2 provided evidence that corroborates the idea of such reasoning under the assumption of truth. The results further suggest that especially people with higher cognitive ability seem to be susceptible to spontaneously exhibiting such deductively rational reasoning.

## Appendix A

Table A presents items used in Experiments 1 and 2 and their grouping as items with relatively few versus many exceptions and/or alternatives. The item characteristics are not those obtained by the pre-tests conducted on these Dutch stimuli by De Neys et al. (2003) or Verschueren et al. (2005). They represent the results of a new validation study in the same population Experiment 1 and 2 was run in. That is, 11th-12th grade students (*n* = 21) generated alternatives and another group of 26 students of the same high-school generated exceptions to the rules. The alternatives and exceptions questionnaire was identical to the one used by Verschueren et al. That is, participants were instructed to generate up to five conceptually distinct alternatives or exceptions and were asked to rate the likelihood of each of the alternatives (alternatively exceptions) on a 7-point scale going from 1 (*occurs almost never*) to 7 (*occurs very often*) over 4 (*occurs sometimes*). Salience is computed as the proportion of subjects who generated the most frequently generated alternative or exception.

**Table A.** Average Number of Alternatives and Exceptions (Frequency [F]), Their Average Plausibility (P) and Salience (S), and Grouping (G) as Items With Relatively Few Versus Many Exceptions and/or Alternatives.

	Exceptions				Alternatives			
	G	F	P	S	G	F	P	S
If the match is struck, then it lights.	Many	2,10	4,98	55,6	Few	1,33	4,17	100
If the correct switch is flipped, then the porch light goes on.	Many	2,62	4,58	90,0	Few	1,74	4,04	25,0
If water is heated to 100°C, then it boils.	Few	0,76	4,27	33,3	Few	1,22	3,65	21,1
If one cuts ones finger, then one bleeds.	Few	1,57	4,68	50,0	Few	1,91	4,61	28,6
If fertilizer is put on plants, then they grow quickly.	Many	2,14	4,78	52,6	Many	1,83	4,95	33,3
If one turns on the air conditioner, then one feels cool.	Many	2,14	4,75	55,0	Many	1,96	4,65	38,1
If one jumps into the swimming pool, then one gets wet.	Few	1,76	4,37	94,4	Many	2,30	5,22	68,2
If the apples are ripe, then they fall from the tree.	Few	1,60	3,63	26,3	Many	2,05	4,41	57,1

## Appendix B

Appendix B presents the basic certainty ratings of the logically valid (MP, MT) and logically invalid (AC, DA) arguments about conditionals with few or many alternatives and/or exceptions (a.k.a. *disablers*) observed in the standard designs used in Experiments 1 and 2. All certainty ratings were obtained by trans- forming the selected evaluation on the 5-point or 7-point evaluation scales to the uniform [0,1] probability intervals.

**Table B1.** Basic Certainty Ratings as a Function of Few or Many Exceptions and/or Alternatives to the Conditional Relation (Experiment 1, ***N*** = 100).

	Alternatives			
	Many exceptions		Few exceptions	
Argument	Many	Few	Many	Few
MP	0,741	0,877	0,748	0,922
AC	0,552	0,434	0,762	0,847
MT	0,559	0,705	0,531	0,814
DA	0,530	0,412	0,792	0,860

**Table B2.** Basic Certainty Ratings as a Function of the Frequency of Exceptions and/or Alternatives (Experiment 2).

	Many exceptions				Many exceptions				Few exceptions				Few exceptions			
	Many alternatives				Few alternatives				Many alternatives				Few alternatives			
	Argument				Argument				Argument				Argument			
Instruction	MP	MT	AC	DA	MP	MT	AC	DA	MP	MT	AC	DA	MP	MT	AC	DA
Standard (*n* = 129)	.793	.526	.492	.436	.763	.671	.789	.796	.847	.605	.499	.458	.870	.786	.878	.833
Speeded (*n* = 116)	.763	.517	.467	.415	.739	.678	.773	.796	.813	.540	.504	.478	.838	.772	.863	.833
Mean	.779	.522	.480	.426	.752	.674	.781	.796	.831	.574	.502	.468	.855	.779	.871	.833
